# Spiritual Well-Being, Social Support, and Financial Distress in Determining Depression: The Mediating Role of Impact of Event During COVID-19 Pandemic in Iran

**DOI:** 10.3389/fpsyt.2021.754831

**Published:** 2021-10-28

**Authors:** Hamid Sharif Nia, Ozkan Gorgulu, Navaz Naghavi, María Auxiliadora Robles-Bello, David Sánchez-Teruel, Fatemeh Khoshnavay Fomani, Long She, Pardis Rahmatpour, Kelly-Ann Allen, Gokmen Arslan, Saeed Pahlevan Sharif

**Affiliations:** ^1^Psychiatry and Behavioral Sciences Research Center, Addiction Institute, Mazandaran University of Medical Sciences, Sari, Iran; ^2^Department of Biostatistics and Medical Information, Faculty of Medicine, Kirsehir Ahi Evran University, Kirsehir, Turkey; ^3^Taylor's Business School, Taylor's University, Subang Jaya, Malaysia; ^4^Area of Developmental and Educational Psychology, Department of Psychology, Faculty of Humanities, University of Jaén, Jaén, Spain; ^5^Department of Personality, Evaluation and Psychological Treatment, Faculty of Psychology, University of Granada, Granada, Spain; ^6^School of Nursing and Midwifery, Tehran University of Medical Sciences, Tehran, Iran; ^7^Faculty of Business and Law, Taylor's University, Subang Jaya, Malaysia; ^8^Department of Nursing, School of Nursing, Alborz University of Medical Sciences, Karaj, Iran; ^9^School of Educational Psychology and Counselling, Faculty of Education, Monash University, Clayton, VIC, Australia; ^10^Centre for Wellbeing Science, The Melbourne Graduate School of Education, The University of Melbourne, Parkville, VIC, Australia; ^11^Mehmet Akif Ersoy University, Burdur, Turkey; ^12^Faculty of Business and Law, Taylor's University, Subang Jaya, Malaysia

**Keywords:** COVID-19, depression, financial distress, Iran, social support, spiritual well-being, stress

## Abstract

This study investigates the relationship between spiritual well-being, social support, and financial distress with depressive symptoms due to the COVID-19 pandemic. A path analysis was used to analyze data collected from 1,156 Iranian participants via an online survey. The results showed that spiritual well-being and social support were negatively related to depressive symptoms and financial distress. The impact of COVID-19 events showed negative associations with depressive symptoms. In addition, the link between spiritual well-being and financial distress with depressive symptoms was partially mediated by the impact of events.

## Introduction

The unprecedented rate of mortality from the coronavirus (COVID-19) pandemic has caused times of distress and uncertainty for people across the globe (1–4). Iran has not been an exception. At the time of writing this paper (16 July 2021), Iran had 3,440,400 confirmed cases and 86,391 virus-related deaths since the beginning of the pandemic (5).

Restrictions to curb the spread of the COVID-19 virus, such as quarantine measures, stay-at-home recommendations, physical distancing orders, as well as the fear of potential exposure to infection and misinformation, have taken a heavy toll on public psychological well-being (6). The effects of the pandemic have disrupted not only peoples' everyday lives but also reduced social connections and a sense of belonging that people would usually experience (7–9). Such outcomes are strongly linked to depression (3, 10–12). In addition, the economic recession due to lockdowns, an increase in unemployment (13), and uncertainty about the affordability of health costs (14) may lead to stressors that trigger depression and other mental disorders (15).

It has been reported that anxiety and depression due to COVID-19 have had a prevalence rate between 16 and 20% across the general population in various countries (16). Specifically, findings from a meta-analysis suggested a significant difference in the general population between global depression rates in 2017 (3.44%) and 2020 (25%) (17).

### Literature Review

Many factors have been put forth in the body of literature focusing on the determinants of depression among the public during the pandemic. The first strand of literature sheds light on various types of self-care due to physical and social constraints (18), out of which spirituality has drawn growing attention (18–20). The underpinning theory for this category of literature is the *mindful consumption* notion proposed by Sheth et al. (21), which discusses the human capability to develop resilient solutions in times of hardship. From this point of view, spirituality is an intrinsic motivation to seek satisfaction and maintain a harmonious relationship with oneself/others in times of stress or crisis (20–23). Studies that have examined spirituality during COVID-19 have found that it can facilitate self-reported self-care, connectedness, and meaning and purpose in life (18, 24, 25).

Given this, spiritual dimensions have been integrated into research on coping, well-being, and mental health promotion across the lifespan, as a way to prevent mental health disorders such as depression (26–29). The impact of COVID-19 on spirituality has been assessed by some literature, which found that a higher perceived COVID-19 risk predicted more significant depressive symptoms (30). In most cases, when people perceive a stressful, aversive, or traumatic event, such as a pandemic they may engage in religious activities or rituals to cope with depressive symptoms (31–33). Although the most literature has found a positive relationship between spirituality and mental health (34), some research has reported a negative or neutral relationship (4, 35).

Another strand of literature highlights the importance of social support as a psychosocial protective factor concerning mental health adversity (36–39). Social support is the subjective evaluation of friends', family members', and significant others' ability to provide support in challenging times (40). The stress-buffering hypothesis (41) posited that interpersonal social support could buffer the negative impact of hardship and enhance an individual's ability to reduces stress, anxiety, and depression (42–44). There is a plethora of research on the negative association between social support and severity of mental health outcomes, such as depression and anxiety (43, 45, 46). The vital role of social support has been highlighted during the pandemic (47–49) with some studies finding the impact of COVID-19 improved individuals' perceived social support (50). Yu et al. (51) found that during the COVID-19 pandemic, participants with the highest amount of psychological distress received the least amount of social support. They also displayed more passive coping styles compared to participants with lower distress. Along these lines, the current study seeks to investigate the relationship between social support and depressive symptoms during the pandemic among the Iranian public.

Apart from introspective (e.g., spiritual well-being) or extroverted (e.g., social support) factors, the financial burden caused by the pandemic is another influential factor related to mental health issues. Economic burden from the pandemic includes both direct costs (e.g., virus-related medical treatment) and indirect costs (e.g., job loss) (52, 53). The enforced lockdowns of many businesses to control the infection rate have caused increased unemployment in many sectors. The high transmission rate of the virus has inflicted medical costs, including those from diagnosis, treatment, and hospitalization (if required) to many infected individuals and families. Such financial pressures may cause stressors that can contribute to depression.

Although the relationship between perceived financial burden and depression in patients with chronic disease has been extensively investigated in the literature (54–57), limited research has investigated this during the COVID-19 pandemic. A few recent studies have affirmed perceived financial burden as an emerging worrying factor for some specific groups of people during the pandemic (58, 59). There is, however, a need for a detailed investigation into this relationship by examining the possible variables that may affect financial burden and depression in different settings.

To ensure the current study captures the impact of the pandemic on individuals' mental health, event-specific distress is taken into consideration. The *Impact of Event Scale-Revised* used in this study measures stress or distress by evaluating: Intrusions (unwanted thoughts and images related to the event such as nightmares), Avoidance (the effort to avoid thinking about the traumatic or stressful event), and Hyperarousal (anger, irritability, difficulty concentrating) [Hair (60–62)]. This scale has been widely used in literature to measure COVID-19's impact on individuals' stress levels (63–67).

According to a review conducted by Rajkumar (16), only eight publications have explored mental health problems as influenced by the COVID-19 pandemic in the general population. While only a few studies have focused on the prevalence of anxiety and depression among healthcare professionals (68–71) and COVID-19-infected patients (72) since the start of the pandemic. Limited attention has been given to the public. Moreover, to date, factors influencing depression and depressive symptoms among the general population remain largely unknown (38, 73). Therefore, the current study investigates some factors related to depressive symptoms in the general population due to the pandemic. Moreover, this study goes one step further to investigate the mediating role of stress related to the impact of the pandemic (i.e., “impact of the event”) on the relationships between spiritual well-being, financial distress, and social support with depression. In this vein, the current study aims to analyze the direct relationship between spiritual well-being, social support, and financial distress with depressive symptoms and endeavors to explore if there are any indirect relationships among the aforementioned variables with the impact of the event as a mediator.

This study intends to investigate the factors influencing depression due to the COVID-19 pandemic, which will provide policymakers insight into what factors and approaches could be prioritized. The noteworthy contributions of the present study are 2-fold. To the best of the authors' knowledge, it is the first study considering the indirect relationship between spiritual well-being (SWB), social support, and financial distress with depressive symptoms during the pandemic. Secondly, owing to the widespread impact of the pandemic, this study surveys the general population in Iran.

### Hypotheses

Seven hypotheses were investigated in the current study:

H_1_: Social support will have a significant negative correlation with impact of event, and social support will be a significant predictor of impact of event.
H_2_: Social support will have a significant negative correlation with depression, and social support will be a significant predictor of depression.
H_3_: Spiritual well-being will have a significant negative correlation with impact of event, and spiritual well-being will be a significant predictor of impact of event.
H_4_: Spiritual well-being will have a significant negative correlation with impact of event, and spiritual well-being will be a significant predictor of depression.
H_5_: Financial distress will have a significant positive correlation with impact of event, and financial distress will be a significant predictor of impact of event.
H_6_: Financial distress will have a significant positive correlation with depression, and financial distress will be a significant predictor of depression.
H_7_: Impact of event will have a significant positive correlation with depression, and impact of event will be a significant predictor of depression.

## Method

A predictive, cross-sectional online questionnaire-based survey was used in this study to investigate the relationships between spiritual well-being, social support, and financial distress with depressive symptoms due to the pandemic and the mediating role of the impact of the COVID-19 event in these relationships.

### Participants

The requisite sample size was estimated to be 1,156 with a probability of 0.05, the statistical power of 80%, the anticipated medium effect size of 0.13, and 64 items measuring five constructs. This estimate was calculated a-priori using a sample size calculator for Structural Equation Models (SEM) (74). The minimum statistical power analysis in humanities and social sciences studies should be 80% (75). In total, 1,156 participants in Iran participated between April and July 2020 during the initial stages of the COVID-19 pandemic. The questionnaire was designed with Google Form and was made available to the public through Telegram channels and WhatsApp public groups. The mean age of participants was 32.78 (SD = 7.7) (range 20 to 60) years old, and most were female (83.5%), married (51.3%), and had a bachelor's degree (48.1%). The other socio-demographic information is provided in Table 1.

**Table 1:** Demographic characteristics of participants (*n* = 1,156).

**Variables**	***N* (%)**
**Gender**	
Female	965 (83.5)
Male	191 (16.5)
**Marital status**	
Single	563 (48.7)
Married	593 (51.3)
**Education level**	
Under diploma	25 (2.2)
Diploma	118 (10.2)
Bachelor	556 (48.1)
Master/PhD	457 (39.5)
**Employment**	
Unemployed	333 (28.8)
Employed	605 (52.3)
Student	218 (18.9)
**History of COVID-19**	
Yes	138 (12.1)
No	1,003 (87.9)
**Family history of COVID-19**	
Yes	284 (24.9)
No	857 (75.1)
**Variables (Range of scale score)**	**Mean ± SD**
Depression (1–48)	37.70 ± 9.5
Financial distress (11–55)	36.42 ± 6.1
Social support (9–54)	21.54 ± 7.4
Impact of event (0–88)	35.6 ± 17.2
Spiritual well-being (1–55)	32.18 ± 13.8

### Instruments

A demographic form and the Persian version of the following scales were used in this study: ENRICHD Social Support Inventory (ESSI), the Spiritual Well-Being (SWB) Scale, the Comprehensive Score for Financial Toxicity (COST), the Center for Epidemiological Studies Depression scale (CES-D), and the Impact of Events Scale-Revised (IES-R).

The Persian version of ENRICHD Social Support Inventory (ESSI) consists of six questions and utilizes a five-point Likert scale, ranging from 1 (*none of the time*) to 5 (*all of the time*) (76). Construct validity of the Persian scale was conducted by maximum likelihood exploratory factor analysis. The results extracted one factors accounting for 64.307% of the variance. The reliability of the ESSI was determined by Cronbach's alpha and McDonald's omega, which were found to be 0.91.

The Persian version of the SWB Scale (27) consists of two subscales: connecting with God and meaningless life. Only the subscale “connecting with God” (eight items) was used in the current study. The items were measured using a 6-point Likert-type scale from 1 (completely disagree) to 6 (completely agree). A higher score indicated greater spiritual well-being or greater connection with God in the present study. The reliability of the SWB Scale was determined by Cronbach's alpha and McDonald's omega, which were found to be 0.911 (CI 95%: 0.904 to 0.917) and 0.935 (CI 95%: 0.930 to 0.941), respectively.

The Persian version of the Comprehensive Score for Financial Toxicity (COST) scale (77) contains 11 questions is scored from 1 (strongly disagree) to 5 (strongly agree) (78). The reliability of the COST scale was determined by Cronbach's alpha and McDonald's omega, which were found to be 0.820 (CI 95%: 0.804 to 0.835) and 0.823 (CI 95%: 0.808 to 0.838), respectively.

The Persian version of the center for epidemiological studies depression scale (CES-D) was used to measure symptoms associated with depression. It consists of 16 items with three factors, namely somatic affect (7 items), negative affect (5 items), and positive affect (4 items), with a four-point Likert response (79). The reliability of the CES-D was determined by Cronbach's alpha and McDonald's omega, which were found to be 0.874 (CI 95%: 0.864 to 0.884) and 0.883 (CI 95%: 0.874 to 0.893), respectively.

The Persian version of the Impact of Events Scale-Revised (IES-R) consists of 18 items and three factors that measure different dimensions of stress response, including intrusion (6 items), avoidance (7 items), and hyperarousal (5 items). The IES-R is a short, easily administered scale that can be used with most individuals exposed to any specific traumatic event. The IES-R is scored on a 5-point Likert-type scale from 0 (not at all) to 4 (extremely) (61). The reliability of the IES-R was determined by Cronbach's alpha and McDonald's omega, which were found to be 0.930 (CI 95%: 0.924 to 0.936) and 0.929 (CI 95%: 0.923 to 0.935), respectively.

### Data Analysis

A path analysis was used in the present study. It is used to determine how much of the total effects of independent variables on the dependent variables occur directly and indirectly. The method of path analysis was originated and developed by the geneticist Sewall Wright as early as 1918 (80, 81). Path analysis uses multiple regression techniques that allow a second dimension-time sequence to enter the analysis (82). As used in this study, path analysis utilizes a standard multiple regression technique to estimate the path coefficients. The standard regression coefficients from the multiple regressions were the path coefficient (83). In path analysis, four different effects as direct, indirect, U, and S between variables are defined.

Statistical Package for Social Sciences (version 25) for Windows (IBM SPSS Statistics for Windows, Version 21.0; IBM Corp., Armonk, NY) was used for the analyses in the study. JASP 14.0.0 was also used to evaluate McDonald's omega. A probability level of *p* < 0.05 was used to determine statistical significance.

### Ethical Considerations

The study aims, number of items, time to complete the survey, the researcher's affiliation and email for queries, and the ethical code of study were inserted on the first page of the online questionnaire. These items informed participants that their participation was voluntary and that their responses would be published anonymously as group data. The protocol of this study was approved by the Mazandaran University of Medical Sciences Research Ethics Committee (IR.MAZUMS.REC.1399.7461).

## Results

**Figure 1:**
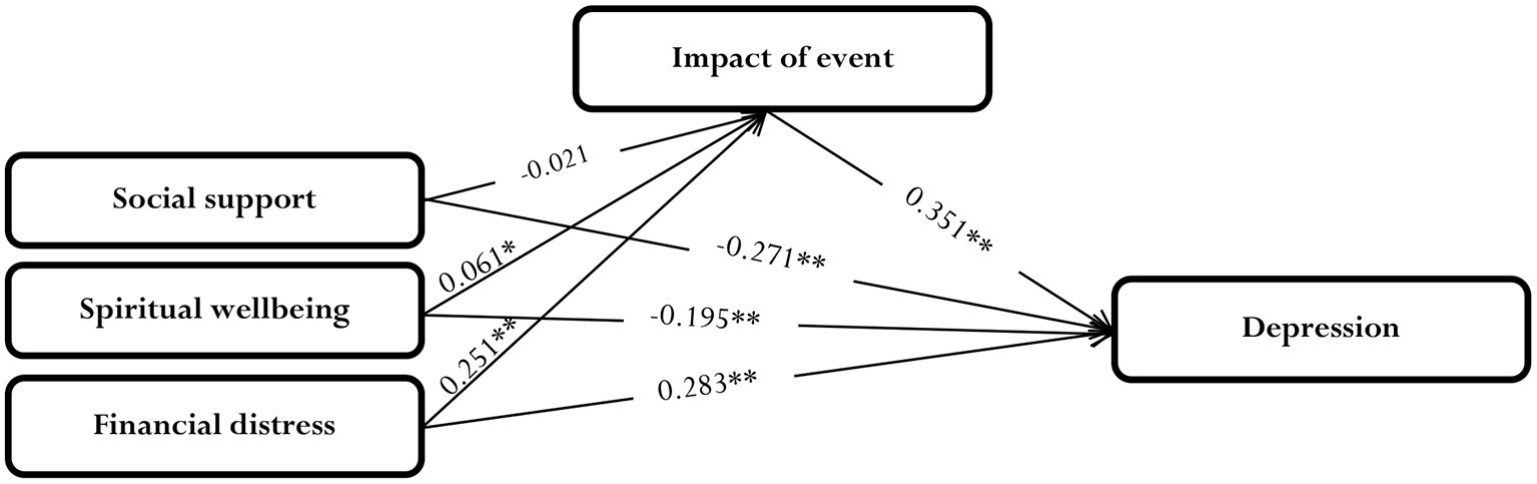
Path diagram. **p < 0.01, *p < 0.05.

According to the path diagram (Figure 1) and the results of the path analysis (**Table 3**), this study revealed that the total effects of social support on depression (total effect = −0.378^**^, *p* < 0.01), and spiritual well-being on depression (total effect = –0.357^**^, *p* < 0.01) were negative and significant. In addition, the results indicated that the total effect of financial distress on depression (total effect = *0.4*73^**^, *p* < 0.01), and impact of event on depression were positive and significant (total effect = *0.4*16^**^, *p* < 0.01).

The correlation matrix displaying the relationships between depression and other variables is given in Table 2. There was a significant positive relationship between financial distress and impact of event with depression (r = 0.469^**^, r = 0.430^**^, *p* < 0.01). The relationships between social support and spiritual well-being with depression were significant and negative (r = −0.389^**^, r = −0.344^**^, *p* < 0.01). The correlations between social support, spiritual well-being, and financial distress variables were positive and statistically significant (*p* < 0.01). These correlations created the unanalyzed effect in the model (see Figure 1).

**Table 2:** Correlations between variables.

	**Social support (X1)**	**Spiritual well-being (X2)**	**Financial distress (X3)**	**Impact of event (X4)**	**Depression (Y)**
Social support (X1)	1				
Spiritual well-being (X2)	0.240^**^	1			
Financial distress (X3)	−0.186^**^	−0.267^**^	1		
Impact of event (X4)	−0.054	−0.009	0.233^**^	1	
Depression (Y)	−0.389^**^	−0.344^**^	0.469^**^	0.430^**^	1

** *p < 0.01*.

The coefficients, which show the direct and indirect effects of the variables on depression, are summarized in Table 3. According to these results, social support had a negative significant direct effect on depression (P_YX1_ = −0.271^**^, *p* < 0.01). Social support indirectly affected depression through spiritual well-being and financial distress. The ratio of the U effects of the social support variable on spiritual well-being and financial distress were 12.08 and 13.62% within the total effect, respectively.

**Table 3:** Path effects.

**Pathways**	**Effect value**	**%**
**Social support** ***(X1)—*****depression** ***(Y)***		
Direct effect	−0.271^**^	69.67
Indirect effect	−0.007	1.80
U effect (X2)	−0.047	12.08
U Effect (X3)	−0.053	13.62
*Total effect*	*−0.378* ^**^	*97.17*
*Error*	*0.011*	*2.83*
*Total correlation*	−0.389^**^	*100*
**Spiritual well-being** ***(X2)—*****depression** ***(Y)***		
Direct effect	−0.195^**^	56.69
Indirect effect (X4)	−0.021^*^	6.10
U Effect (X1)	−0.065	18.90
U Effect (X3)	−0.076	22.09
*Total effect*	*−0.357* ^**^	*103.78*
*Error*	*0.013*	*−3.78*
*Total correlation*	−0.344^**^	*100*
**financial distress** ***(X3)—*****depression** ***(Y)***		
Direct effect	0.283^**^	60.34
Indirect effect (X4)	0.088^**^	18.76
U Effect (X1)	0.050	10.66
U Effect (X2)	0.052	11.09
*Total effect*	*0.473* ^**^	*100.8*
*Error*	*−0.004*	*−0.02*
*Total correlation*	0.469^**^	*100*
**Impact of event (X4)—depression** ***(Y)***		
Direct effect	0.351^**^	81.63
Spurious Effect (X1)	0.006	1.40
Spurious Effect (X2)	−0.012	−2.79
Spurious Effect (X3)	0.071	16.51
*Total effect*	*0.416* ^**^	*96.74*
*Error*	*0.014*	*3.26*
*Total correlation*	0.430^**^	*100*

** *p < 0.01,*

* *p <0.05. X = independent variable.*

*Y = dependent variable*.

The direct effect of the spiritual well-being variable (P_YX2_ = −0.195^**^, p < 0.01) on depression constituted 56.69% of the total effect. The U effect of spiritual well-being on financial distress accounted for 22.09% of the total correlation. The direct effect of financial distress on depression was quite high (P_YX3_ = 0.283^**^, p < 0.01, 60.34%). The indirect effect on impact of event was the highest (18.76%). When the variables are examined in terms of direct effects, it is seen that the highest effect is calculated in the impact of event variable. Indirect effects can be interpreted using the mediator variable. The mediator variable in this study was impact of event as can be seen in the model and path diagram. The effects of the social support, spiritual well-being, and financial distress variables on depression through impact of event provided the indirect effects in the framework of the model. The results showed that effects of social support (−0.007, *p* = 0.46), spiritual well-being (−0.021^*^ *p* < 0.05), and financial distress (0.088^**^, *p* < 0.0.01) on depression through impact of event demonstrated that impact of event mediated the relationship between spiritual well-being and depression, and between financial distress and depression, but not the relationship between social support and depression. Moreover, the significant direct effects of spiritual well-being (P_YX2_ = −0.195^**^, p < 0.01) and financial distress (P_YX3_ = 0.283^**^, p < 0.01) on depression in the mediation model indicated both mediation effects were partial. Lastly, impact of event had a strong and positive direct effect on depression (P_*yx*4_ = 0.351^**^, *p* < 0.01, 81.63%). In addition to the direct effect of the impact of event variable, it also significantly (16.51%) spurious effect on depression of financial distress variable.

## Discussion

The current study investigated certain factors that associated with depressive symptoms as reported by Iranians during the COVID-19 pandemic. More specifically, this study examined how social support, spiritual well-being, and financial distress predicted depression among the general population in Iran during the COVID-19 pandemic.

The findings revealed that there was a significant positive correlation between financial distress and the impact of events (e.g., quarantine during COVID-19) with depressive symptoms. Global economic instability, changes in employment, and fear of future financial outcomes due to the COVID-19 pandemic are some possible causes of financial distress among individuals (84). Many businesses were forced to shut down, and some small businesses closed permanently. While overcoming economic upheaval is helped markedly by an individuals' financial standing (85), COVID-19 affected such a large number of people with varying financial needs and capacities that financial distress for many was unavoidable. The positive correlation between financial distress and depressive symptoms has been identified in several studies [see (86–88)]. There is also evidence of increased suicide rates (89), aggression, and litigation (90) during previous quarantine periods. Furthermore, gender differences have been found in terms of mental health consequences during the COVID-19 outbreak. Females may experience more severe stress, depression, and anxiety symptoms during the COVID-19 (91–93). As most of the participants (83.5%) in the present study were female, the relationship between the impact of event (e.g., quarantine) and depressive symptoms may be overemphasized.

Quarantine is an unpleasant experience for many, in part due to a loss of freedom, uncertainty over the virus, and separation from close social contacts (94). In the COVID-19 pandemic, restrictions due to lockdown measures, a reduction of social connections, and greater perceived uncertainty have been identified as variables associated with greater mental health problems and depressive symptoms (8). Consistent with past studies (38, 43), the current study findings revealed a negative relationship between social support and depressive symptoms. However, the exact relationship between depression and social support has varied in the literature. While some study findings have found a negative relationship between these variables (95), others have found differences between the support offered by family, neighbors, or friends with social support from the family having a more substantial effect on mental health (96). Social support has been found to buffer the effect of stress on depression (97) through increasing self-disclosure and creating feelings of safety (98). Torkian et al. (99) reported that a moderate to high number of Iranians received social support during the COVID-19 outbreak, which improved their social adjustment. One explanation for the current findings could be that social support facilitates social adjustment to change and uncertainty, which mitigates the risk of depression.

Congruent with past studies (100–102), the results indicated that there is a negative relationship between spiritual well-being and depressive symptoms, which has been supported by past research (100, 101, 103). Few studies have focused on the relationship between religion and depression in the Iranian population. The existing literature indicates there is a negative association between religiosity and depression among medical students (104), Iranian patients with spinal cord injury (105), and infertile women (106). A qualitative systematic review of 4,944 papers provided evidence of the significant role of spirituality in improving mental health, namely that spirituality gives meaning to life. It improves coping skills that may help mitigate depressive symptoms (34). Furthermore, a study with a sample of Palestinian adults during the spread of COVID-19 found a negative correlation between positive religious coping and depressive symptoms (35). Similarly, the findings of a study with an Arab population showed negative religious coping was associated with depression (4). However, a reverse relationship between spirituality and depression has been demonstrated by several other study findings (107). While religious beliefs and practices may help people to cope better with life adversities via giving their life meaning and hope, in some cases, religious beliefs may also increase feelings of guilt and failure (108).

One important consideration when interpreting the relationship between religion and mental health is the context. One study compared 268 regions within 28 European countries and found that individuals from a religious minority reported more depressive symptoms than individuals from non-minorities, except in regions where there is a majority of Muslims (109). Another study showed that the mental health of Iranian people is driven by extrinsic religious motivation more than people living in Western countries (110). Therefore, when Iranian people cannot attend religious places and perform group ceremonies or rituals due to quarantine, their mental health may be more affected (110). It is worth mentioning that women tend to participate more frequently than men in religious practices, which may have influenced the present study's findings (111, 112). The development of online spiritual health programs for the Iranian population during times of lockdown may have merit in the future.

The current study revealed that spiritual well-being and financial distress had significant indirect relationships with depressive symptoms through the different dimensions of stress response caused by the impact of event (e.g., COVID-19). Among these variables, financial distress and the impact of event had the highest direct and indirect effect with depressive symptoms, respectively. The virus outbreak and the quarantine that followed may have led to widespread stress, especially stigmatization and social exclusion, which may escalate into other negative psychological reactions, including adjustment disorder and depression (113). During the COVID-19 pandemic, many people went through financial hardship due to the increasing cost of healthcare expenditures (114). Furthermore, the COVID-19 pandemic resulted in an unprecedented decline in economic activity with employment and a decline in income (100). Despite the government's protective measures to reduce peoples' anxiety about the virus, the fear of economic loss has increased mental health problems among Iranian people. While the government launched social support programs and psychological services for patients, it remained incapable of offering economic stimulus packages (115) or proactive and preventative approaches for the general population.

The findings of a large national study in Iran found a high level of stress among the general Iranian population during the COVID-19 outbreak in which those in middle age groups and low to moderate socioeconomic status experienced the highest stress due to worry about losing their jobs or income (116). In particular, middle-income earners (e.g., laborers) experienced stress from their inability to physically attend work. While low-income earners, who attended work, would do so on crowded buses, subways, or other vehicles and experienced stress from the fear of infection or dismissal for non-attendance (117). From these findings, it appears that the COVID-19 pandemic has most negatively affected the socially vulnerable Iranian population. Public policies to protect these groups are essential to minimize the spread of COVID-19 in the country and prevent the development of clinical disorders such as depression.

The impact of the COVID-19 events significantly mediated the relationship between spiritual well-being and depression. Although spiritual well-being has been identified as a protective factor for depression during the COVID-19 outbreak (118), the impact of religion on better mental health outcomes is dependent on the number of religious activities engaged in, physical religious attendance, and increased spiritual growth (33, 119). Since quarantine began in Iran, many public spiritual or religious activities were ceased due to mosques and religious places closing. Given that the majority of Iranians are Muslim, public spiritual activities such as congregational prayer have long been an important practice for them. Accordingly, it is plausible that Iranians barred from attending public religious practice may feel psychological distress. In this regard, the findings of a meta-analysis study that investigated the spirituality of religious effects on mental health revealed that among different religious or spiritual factors, only participation in public religious activities and the importance of religion were significantly related to mental health (120).

In general, although the correlations between the current study variables have been addressed by previous studies, the present study revealed the significant positive mediating role of the COVID-19 pandemic on the relationship between spiritual well-being and financial distress with depression. What sets this study apart from previous studies is that it identifies how the effects of the COVID-19 pandemic can affect the relationship between spirituality, financial distress, social support, and the development of depressive symptoms. While many studies suggest that promoting spirituality in times of crisis and epidemics can ensure mental health (33, 121), the present study showed that this is not possible without considering the impact of the event.

### Study Limitation

While the study provides new information relative to the mediating role of the impact of event on the relationship between financial distress and spiritual well-being with depression, it is not without its limitations. The cross-sectional design of this study does not allow for firm causal conclusions. Conducting longitudinal studies by collecting data at different points in time as well as experimental studies are recommended for future research since there are numerous complex and dynamic processes by which spirituality relates to mental health outcomes. In terms of mediation studies, the most salient mediating processes seem to involve religiosity/spirituality dimensions, values/attitudes, and social control/norms, which need to be investigated in further studies. Furthermore, the data were gathered via online data collection. Despite its advantages (e.g., affordability and accessibility), online surveys have been criticized for selection bias and difficulty reaching certain types of participants (122, 123). Finally, the questionnaire length (64 items) and the order of instruments may have affected careless responding (124) or reduced response rate due to response burden (125).

## Conclusion

The current study revealed that during the COVID-19 pandemic, financial distress may have influenced depressive symptoms, however this can be explained through impact of event. In addition, spiritual well-being may not always serve a protective role in terms of the impact of stress on depression. Consequently, when pandemic-related protective measures are rolled out (e.g., quarantine), the positive relationship between spiritual well-being and depression appears. The visibility of protective factors in addition to risk factors can offer a broader view on measures to deal with depression in the general population resulting from global adverse situations such as the ongoing COVID-19 pandemic. The current study findings are applicable for health policy-makers to help them for developing health promotion programs and fostering resilience among the general population. It will also be the responsibility of governments to help improve public health through economic protection policies in the event of epidemics.

## Data Availability Statement

The data that support the findings of this study are available from the corresponding author upon reasonable request.

## Ethics Statement

The studies involving human participants were reviewed and approved by Mazandaran University of Medical Sciences Research Ethics Committee (IR.MAZUMS.REC.1399.7461). The patients/participants provided their written informed consent to participate in this study.

## Author Contributions

PR, HS, and GA contributed to the study conception and design. Material preparation and data collection was performed by FK and PR. OG, HS, and SP performed data analysis. The first draft of the manuscript was written by NN, PR, LS, FK, DS-T, K-AA, and MR-B. All authors commented on previous versions of the manuscript and read and approved the final manuscript.

## Conflict of Interest

The authors declare that the research was conducted in the absence of any commercial or financial relationships that could be construed as a potential conflict of interest.

## Publisher's Note

All claims expressed in this article are solely those of the authors and do not necessarily represent those of their affiliated organizations, or those of the publisher, the editors and the reviewers. Any product that may be evaluated in this article, or claim that may be made by its manufacturer, is not guaranteed or endorsed by the publisher.
